# Annotations

**Published:** 1897-02-27

**Authors:** 


### ANNOTATIONS.

### Sanitation in Health Resorts.

So universal has become the custom of prescribing change of air for convalescents and for all whose ailments tend to resist less radical measures, that it may be considered a matter almost of national concern that our health resorts should themselves be healthy. Some statements recently made in the *Scarborough Post* in regard to the methods of sanitation prevalent in Scarborough are therefore of considerable interest. The lack of proper hospital provision for dealing with infectious cases is a serious drawback to a health resort of such importance. So also is the existence of private slaughter-houses scattered over the town. But the great blot on the sanitary administration is the existence of an enormous number of old-fashioned privies. It is not merely that they exist in the old houses, but their number is being increased, new ones being added yearly to the list, so that now Scarborough, with 8,900 houses, has 6,500 privies! Some of these, it is stated, are in such a position that the contents have to be carried through the house every time the sanitary cart calls. Need we wonder, then, that typhoid fever during the past five years has been more than twice as prevalent as it has been in London? Truly the sanitary evils complained of, and also the typhoid fever, are mostly in the old part of the town, and not in the parts chiefly frequented by visitors. But who shall touch pitch and not be defiled?

### Heroic Doctors.

THAT the study of medicine sometimes attracts men of heroic mould and fibre is proved by the fact that Nansen and Jameson, two of the most heroic men of our generation, are both doctors. That the heroic and enterprising temperament finds itself somwhat "cribbed, cabined, and confined" in medicine is proved by Jameson and Nansen also; for they both left it in order to find in spheres entirely different more adequate scope for energies for which there is little demand in medicine. The profession does not grudge such men to wider, or, at any rate, to very different fields; but it may well wish, for all that, that they had remained loyal to their first love. There is, one cannot help feeling, a lack of heroism and enterprise among those of us who make medicine in one or other of its forms of consulting practice, general practice, or special practice, our life-avocation. It is well worth while trying to find out the cause or causes of this. One cause, undoubtedly, is the tremendous competition in the home profession. The strain of getting into any kind of practice which shall be at once dignified and moderately remunerative, and the equally great, if not greater, strain of keeping unimpaired the modest prize when got, is fatal to enterprise and heroism, and almost to hope. When we have gained a point or points, we plant our feet squarely on the professional ground, as if determined that, come what will, nobody shall move us so much as a single inch from our standing place. Now there can be no heroism here. The changes which are indispensable for the greater efficiency and prosperity of the profession as a whole are never made; they never can be made, for the simple reason that everybody is alike afraid that any movement of any kind, however small, may injure him. We want among us a few men who are not afraid either of their own interests or of professional frowns. Such men, we feel sure, are Nansen and Jameson; and therefore it is that we cannot help wishing they had given their heroism to their original profession instead of to fighting, or to the vanquishing of geographical ignorance in the dark and frozen North.

### Schools and Diphtheria.

A REPORT recently presented by Dr. Hamer to the London County Council on an outbreak of diphtheria in Lewisham which occurred in the autumn of last year, emphasises afresh the important part played by public elementary schools in the dissemination of that fatal disease. The proof lies in a somewhat elaborate tabulation of the cases, the dates of their occurrence, the schools, and the classes of the schools they were attending. Into this we need not go further than to say that there seems every reason for believing that this outbreak was to a large extent the result of school attendance. The type of the disease rapidly acquired a peculiar virulence, nearly half the attacks during the height of the epidemic ending in death. Dr. Hamer suggests that the prevalence of diphtheria would have been more speedily brought to an end by the closing of the schools, and he points to the fact that although they were not actually closed, the parents kept their children away in such large numbers that by October 12th the average attendance in the Board school had fallen to only a little over a third of what it was when it reassembled after the holidays. "During this week the disease practically ceased to manifest a tendency to spread among children attending the Board and National Schools." But to us this does not so much seem to teach the duty of closing the schools as the necessity of not overcrowding them. If with diphtheria rife in the district up to that date, and almost exclusively present among the attendants at these schools, the spread of the disease practically ceased when the attendances fell to a third of what they were before, the moral would seem to be that infection spread through the schools in consequence of overcrowding. This, we take it is the real secret of what is spoken of as "school influence." The children are too tightly packed, and if infection is introduced it spreads from child to child Nothing could be more striking than the distribution by classes in the National School in this case. There were 408 children distributed into 16 classes (including boys, girls, and infants), one of which classes had 23 first sufferers, another 6, another 4, and the remaining 13 none at all. Now, in regard to this it is worth while to recall a remark made by Dr. Felce at a meeting of the Asylums Board about a month ago. Large numbers of patients are every year sent to fever hospitals said to be suffering from diphtheria, who ultimately turn out not to be suffering from that disease. But although sent into the diphtheria wards, they do not, according to Dr. Felce, catch diphtheria, owing probably to the prevalent health conditions. Education we must have. The problem for the school boards is to render attendance in their schools at least as safe as residence in a diphtheria ward! This does not seem much to ask, yet how far from it we seem.

